# The future of heart failure with preserved ejection fraction: Deep phenotyping for targeted therapeutics

**DOI:** 10.1007/s00059-022-05124-8

**Published:** 2022-06-29

**Authors:** Frank R. Heinzel, Sanjiv J. Shah

**Affiliations:** 1 Medizinische Klinik mit Schwerpunkt Kardiologie, Charité – Universitätsmedizin, Campus Virchow-Klinikum, Berlin, Germany; 2 Partner Site Berlin, Deutsches Zentrum für Herz-Kreislauf-Forschung eV, Berlin, Germany; 3 Division of Cardiology, Department of Medicine, Northwestern University Feinberg School of Medicine, Chicago, IL USA

**Keywords:** Cardiovascular disease, Phenotype, Classification, Machine learning, Phenomapping, Herz-Kreislauf-Erkrankungen, Phänotyp, Klassifikation, Maschinelles Lernen, Phänomapping

## Abstract

Heart failure (HF) with preserved ejection fraction (HFpEF) is a multi-organ, systemic syndrome that involves multiple cardiac and extracardiac pathophysiologic abnormalities. Because HFpEF is a heterogeneous syndrome and resistant to a “one-size-fits-all” approach it has proven to be very difficult to treat. For this reason, several research groups have been working on methods for classifying HFpEF and testing targeted therapeutics for the HFpEF subtypes identified. Apart from conventional classification strategies based on comorbidity, etiology, left ventricular remodeling, and hemodynamic subtypes, researchers have been combining deep phenotyping with innovative analytical strategies (e.g., machine learning) to classify HFpEF into therapeutically homogeneous subtypes over the past few years. Despite the growing excitement for such approaches, there are several potential pitfalls to their use, and there is a pressing need to follow up on data-driven HFpEF subtypes in order to determine their underlying mechanisms and molecular basis. Here we provide a framework for understanding the phenotype-based approach to HFpEF by reviewing (1) the historical context of HFpEF; (2) the current HFpEF paradigm of comorbidity-induced inflammation and endothelial dysfunction; (3) various methods of sub-phenotyping HFpEF; (4) comorbidity-based classification and treatment of HFpEF; (5) machine learning approaches to classifying HFpEF; (6) examples from HFpEF clinical trials; and (7) the future of phenomapping (machine learning and other advanced analytics) for the classification of HFpEF.

## Abstract (de)

Die Herzinsuffizienz mit erhaltener Ejektionsfraktion (HFpEF) ist ein systemisches Multiorgansyndrom, das mehrere kardiale und extrakardiale pathophysiologische Anomalien umfasst. HFpEF hat sich als sehr schwierig zu behandeln erwiesen, da es sich um ein heterogenes Syndrom handelt, das einem „One-size-fits-all-Ansatz“ widersteht. Aus diesem Grund haben mehrere Forschungsgruppen an Methoden zur Klassifizierung von HFpEF gearbeitet und zielgerichtete Therapeutika für die identifizierten HFpEF-Subtypen getestet. Neben konventionellen Klassifizierungsstrategien basierend auf Komorbiditäten, Ätiologie, linksventrikulärem Remodeling und hämodynamischen Subtypen wurde in den letzten Jahren eine tiefe Phänotypisierung mit innovativen analytischen Strategien (z. B. maschinelles Lernen) kombiniert, um HFpEF in therapeutisch homogene Subtypen zu stratifizieren. Trotz der wachsenden Begeisterung für solche Ansätze gibt es mehrere potenzielle Fallstricke bei ihrer Verwendung, und es besteht ein dringender Bedarf, datengesteuert identifizierte HFpEF-Subtypen besser zu verstehen, und ihre zugrunde liegenden Mechanismen und molekularen Grundlagen zu erforschen. In der vorliegenden Arbeit wird der Rahmen des phänotypbasierten Ansatzes für HFpEF umrissen: (1) der historische Kontext von HFpEF; (2) das aktuelle HFpEF-Paradigma der komorbiditätsinduzierten Entzündung und endothelialen Dysfunktion; (3) verschiedene Methoden zur Subphänotypisierung von HFpEF; (4) komorbiditätsbasierte Klassifizierung und Behandlung von HFpEF; (5) maschinelle Lernansätze zur Klassifizierung von HFpEF; (6) Beispiele aus klinischen HFpEF-Studien; und (7) die Zukunft des Phänomappings (maschinelles Lernen und andere fortschrittliche Analytik) für die Klassifizierung von HFpEF.

Heart failure (HF) with preserved ejection fraction (HFpEF) is a multi-organ, systemic syndrome that involves multiple cardiac and extra-cardiac pathophysiological abnormalities [1–3]. Because it is a heterogeneous syndrome and resistant to a “one-size-fits-all” approach, HFpEF has proven to be very difficult to treat [4]. By definition, HF is heterogeneous because it is the end result of a wide variety of cardiovascular diseases and risk factors. However, patients with HF with reduced ejection fraction (HFrEF) respond in a more homogeneous manner to therapies [5], whereas HFpEF patients do not. For these reasons, several research groups have been working on combining deep phenotyping with innovative analytical strategies to classify HFpEF into therapeutically homogeneous subtypes [6–11].

Shah et al. initially used a form of machine learning, unsupervised model-based clustering, of deep phenotypic data in HFpEF (*n* = 397), which they termed “phenomapping” [6], and found that from a data-driven perspective, HFpEF is very heterogeneous. In addition, they identified multiple unique HFpEF “pheno-groups” that have differences in clinical characteristics, biomarkers, cardiac structure/function, pathophysiology, and outcomes (validated in a separate group of 107 HFpEF patients). Subsequently there has been a proliferation of such studies throughout the field of HF [12–19].

Despite the novelty of these studies, machine learning analyses [20, 21] are only the starting point for further investigation into HFpEF subtypes, just an initial step on the way to the ultimate goal of unraveling the pathobiology underlying these subtypes so that we can develop effective therapeutics. In addition, at present it is unclear whether machine learning strategies will be the optimal way to identify (1) mechanisms underlying specific types of HFpEF and (2) therapeutically homogeneous HFpEF subtypes. It is likely that a combination of techniques (clinical, pathophysiological, hemodynamic, -omics, exercise testing, and machine learning) will be helpful in the future to identify HFpEF subtypes. Here, we provide a framework for understanding the phenotype-based approach to HFpEF by reviewing (1) the historical context of HFpEF (i.e., diastolic HF); (2) the current paradigm of comorbidity-induced inflammation and endothelial dysfunction as stressors on multiple organs, including the heart; (3) various ways of sub-phenotyping HFpEF, with examples of how these have been leveraged toward potential therapeutics; (4) comorbidity-based classification and treatment of HFpEF; (5) machine learning approaches to classifying HFpEF; (6) examples from HFpEF clinical trials that have informed future phenotype-based HFpEF treatments; and (7) the future of phenomapping (machine learning and other advanced analytics) for classification of HFpEF.

## Historical context of HFpEF

Chronic HF remains a deadly clinical syndrome associated with considerable loss of quality life and high socioeconomic burden. Dyspnea on exertion and exercise intolerance are the leading symptoms but a myriad of clinical manifestations from the early stages of the HF syndrome (e.g., fatigue) to late-stage HF (e.g., loss of appetite, cachexia) can occur. Having emerged as a common and growing type of HF, HFpEF (defined most recently by international consensus as HF with EF ≥ 50% [22]) currently accounts for 50% of all HF and is projected to grow in proportion of HF over the next few decades. Patients with HFpEF generally do not respond to strategies known to improve the prognosis in patients with HF and reduced EF (HFrEF). The reasons proposed for this include a variety of different underlying pathomechanisms (that may not be very responsive to neurohormonal antagonists, in particular), a much greater heterogeneity in therapeutic response, and a higher prevalence of non-cardiac comorbidities compared with HFrEF. It is now clear that HFpEF is not an early form of HFrEF [23]; systematic longitudinal studies have shown that the transition from HFpEF to HFrEF is rare [24]. The lack of prognostic benefit in HFpEF from current HFrEF strategies suggests novel disease mechanisms but also questions the classic concept of myocardial injury as the main driver of disease progression in HFpEF.

In the classic mechanistic concept of HFpEF, the heart was the primary source of the syndrome, with left ventricular (LV) hypertrophy (LVH) and diastolic dysfunction as the main drivers, triggered by systemic hypertension, with contributions from risk factors such as coronary artery disease (CAD). This mechanistic concept arose from early studies in the 1970s of diastolic dysfunction in the cardiac catheterization laboratory, and later by various case series showing impaired filling of the LV in patients with HF and a normal EF [25]. The field was heavily influenced by hypertrophic cardiomyopathy (HCM); indeed, HFpEF was viewed as a forme fruste of HCM in that it was similar pathophysiologically but did not meet criteria for HCM because there was no family history, no genetic abnormalities, and a cause of LVH (hypertension) was present in the majority of patients. The lack of dedicated HFpEF programs also likely impaired this stage of HFpEF understanding because clinicians were not seeing the full extent of HFpEF in the community; rather, it was often patients with severe HFpEF or specific cardiomyopathies (e.g., cardiac amyloidosis, HCM) that were presenting to HF clinics with “diastolic HF” [26]. Furthermore, over the past 50 years, we have witnessed an epidemiological transition from uncontrolled hypertension and smoking (major risk factors for LVH) and high rates of CAD to a better control of these risk factors and an explosion of morbid obesity, diabetes, atrial fibrillation, coupled with an aging population. Whether HFpEF itself has changed due to these epidemiological transitions, or whether it was always the case, it is now clear that HFpEF is not simply cardiac-centric but instead a systemic syndrome, with multiple involved organs; not only the heart but the lungs, liver, adipose tissue, skeletal muscles, and kidneys are all variably involved in individual patients with HFpEF [27].

A primary cardiac insult is the trigger for HFrEF (e.g., myocardial infarction, myocarditis, genetic abnormality, chemotherapy). In HFpEF, however, cardiomyocyte cell death and severe loss of contractility at rest are not commonly observed. Instead, cardiac function in HFpEF is characterized by impaired cardiac filling and altered diastolic properties (stiffness) of the LV, which result in increased LV filling pressure, congestion, and dyspnea on exertion. The underlying pathomechanisms are still not completely resolved, but a leading theory is that myocardial injury in HFpEF is not primary but rather secondary to comorbidity-induced stress on the endothelium [28].

## Comorbidity–inflammation–endothelial dysfunction paradigm of HFpEF

According to a leading conceptual framework of HFpEF, largely based on preclinical disease models, systemic inflammation, triggered by arterial hypertension and metabolic disease states such as diabetes and obesity, leads to coronary microvascular inflammation and dysfunction, subendocardial ischemia, and altered cardiomyocyte mechanics and metabolism (as reviewed in [29]). Thus, HFpEF is considered a result of a multisystem disorder and is strongly associated with advanced age, which may reflect cumulative effects of an increasing number and duration of systemic comorbidities.

## Clinical evidence for heterogeneity in patients classified as HFpEF

The multitude of comorbidities that lead to HFpEF, coupled with the multi-organ, systemic nature of the HFpEF syndrome, contribute to its heterogeneity. Patients with HFpEF share signs and symptoms of HF and echocardiographic features or biomarker evidence of elevated left atrial pressure with preserved LVEF as the main criteria within the current HF classification [30]. However, clinical evidence from prospective trials and observational studies suggests heterogeneity in pathophysiological triggers and clinical presentation with relevance for further diagnostics and therapy. For several decades HCM has been a model for diastolic dysfunction related to structural heart disease, but HCM is found in only a minority (~5%) of HFpEF patients. Based on findings in HCM studies, LVH was considered to be a major contributor to HFpEF because hypertension is a very common comorbidity and has been considered a trigger for HFpEF. Yet, the association between the degree of LVH and diastolic dysfunction in clinical studies is weak, and LVH is only found in about half of the patients with HFpEF [31], which suggests that even myocardial remodeling is heterogeneous in HFpEF.

Recently, HFpEF related to cardiac amyloidosis with accumulation of misfolded proteins in the extracellular matrix has moved into focus, and even though cardiac amyloidosis is likely to be present in a small but relevant fraction of HFpEF patients (~5%, [32]), it is an example of how there may be subtypes of HFpEF that have unique features on cardiac imaging, a specific confirmatory diagnostic approach, and targeted therapy. The common form of HFpEF has been associated with metabolic disease such as obesity or diabetes. Indeed, seminal studies in human myocardium from HFpEF patients have suggested that diabetes mellitus, which is present in almost half of HFpEF patients (45%, [33]), may induce specific alterations in myocardial passive stiffness not observed in non-diabetic HFpEF tissue [34, 35]. In this context, HFpEF is now considered one manifestation of diabetic cardiomyopathy [36] with a higher prevalence of myocardial hypertrophy and fibrosis and a worse prognosis. Chronic kidney disease (CKD; defined as estimated glomerular filtration rate < 60 ml/min/1.73 m^2^), present in approximately 50% of HFpEF patients [37], may be another discriminator. For instance, in a recent Japanese study, patients with moderate CKD but not patients with manifest LVH profited from renin–angiotensin–aldosterone system inhibitors [38]. Varying definitions for the cut-off for a preserved EF in randomized trials (between 40 and 50%) have added to the perceived variability also between HFpEF cohorts. For instance, the fraction of patients with ischemic heart disease was higher in studies with a lower EF cut-off (≥ 40%) as in the CHARM-Preserved (65% of patients) and EMPEROR-Preserved (36% of patients) trials compared with studies with a higher EF cut-off (≥ 45%) as in the PARAGON (23% of patients with ischemic heart disease) and PARAMOUNT (21% of patients) trials [39–42].

## Methods for classification of HFpEF

A variety of methods for classification of HFpEF have been proposed, and each may be useful clinically for selecting appropriate therapies and designing targeted clinical trials. These include clinical (etiological, or primary comorbidity driving HFpEF in a particular patient), pathophysiological (primary pathophysiology driving HFpEF in a particular patient), myocardial, type of clinical presentation, and data-driven approaches to the classification of HFpEF (Table 1). Examples of methods for identification of specific HFpEF subtypes, and how these strategies can lead to novel therapies, are discussed next.

**Table 1:** Classification schemes for heart failure with preserved ejection fraction (HFpEF). Reproduced with permission from [82]

Classification scheme	Categories of HFpEF	Description
Clinical classification	“Garden-variety” HFpEF	HTN, diabetes, obesity, and/or chronic kidney disease
	CAD-HFpEF	Typically, multivessel CAD with prior coronary revascularization
	Right heart failure-HFpEF	Predominant right-sided HF with or without pulmonary HTN
	Atrial fibrillation-predominant HFpEF	Atrial arrhythmias dominate the clinical presentation
	HCM-like HFpEF	These patients do not have genetic forms of HCM, but their clinical course and echocardiographic features are typical of HCM
	High-output HFpEF	Typically, due to liver disease, severe anemia
	Valvular HFpEF	Multiple moderate valvular lesions
	Rare causes of HFpEF	For example, infiltrative cardiomyopathies, cardiotoxicities, genetic cardiomyopathies
Presentation phenotypes	Exercise-induced increase in LA pressure	These patients typically are very breathless with exertion but do not have overt signs of volume overload and typically do not have a history of HF hospitalization
	Volume overload	Signs and symptoms of volume overload; typically have a history of HF hospitalization
	RV failure + pulmonary HTN	Right heart failure predominates the clinical picture; often pulmonary HTN is present and systemic blood pressure is reduced
Myocardial phenotypes	Type 1: HCM	Typical genetic forms of HCM
	Type 2: Infiltrative	Cardiac amyloidosis and other forms of infiltrative or restrictive cardiomyopathies
	Type 3: Non-HTN, non-LVH	No history of HTN and LV wall thickness < 1.2 cm
	Type 4: HTN	Typical, “garden-variety” form of HFpEF with history of HTN
Latent class analysis	A: Younger males with CAD, lower LVEF	Based on latent class analysis of the I‑PRESERVE AND CHARM-Preserved trials. The authors used latent class analysis of 11 clinical features (age, gender, BMI, atrial fibrillation, CAD, diabetes, hyperlipidemia, valvular disease, alcohol use, eGFR, and hematocrit) to find 6 distinct groups of HFpEF in I‑PRESERVE and validated these findings in CHARM-Preserved
	B: Younger females with lowest NT-proBNP	
	C: Obesity, hyperlipidemia, diabetes mellitus, anemia, and renal insufficiency	
	D: Obese females	
	E: Older males with CAD, lowest LVEF	
	F: female predominance, advanced age, lower BMI, atrial fibrillation, CKD, highest NT-proBNP	
Phenomapping	Pheno-group 1: BNP deficiency syndrome	Model-based clustering of 67 continuous variables (phenotypes): physical characteristics, vital signs, ECG data, laboratory data, and echocardiographic parameters
	Pheno-group 2: Cardiometabolic phenotype	
	Pheno-group 3: RV failure + cardiorenal phenotype	

*CAD* coronary artery disease, *HCM* hypertrophic cardiomyopathy, *HF* heart failure, *RV* right ventricular, *HTN* hypertension, *LVH* left ventricular hypertrophy, *LVEF* left ventricular ejection fraction, *BMI* body mass index, *CKD* chronic kidney disease, *NT-proBNP* N-terminal pro-B-type natriuretic peptide

### Clinical subtype: left atrial myopathy

**Fig. 1:**
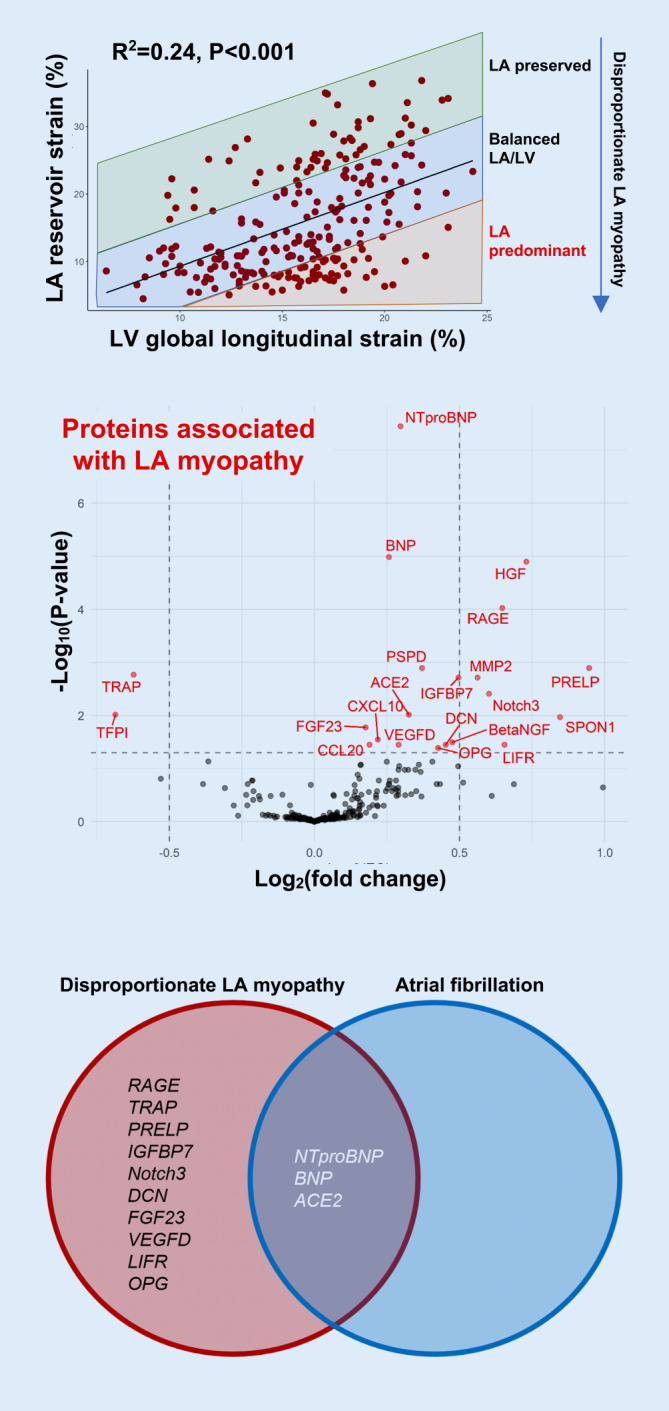
Left atrial myopathy phenotype of heart failure with preserved ejection fraction (HFpEF). *Top panel*: Scatterplot of left atrial (*LA*) vs. left ventricular (*LV*) longitudinal strain showing a correlation between the two phenotypes and deviation from the correlation representing different LA/LV phenotypes of HFpEF, including the “LA predominant” (i.e., LA myopathy) phenotype, defined as LA reservoir strain lower than expected for any given value of LV longitudinal strain. *Middle panel*: Volcano plot of proteins associated with LA myopathy. Proteins *in red on the right* represent those in which higher levels are significantly associated with increased LA myopathy, and *on the left* represent those in which higher levels are significantly associated with reduced LA myopathy. *Bottom panel*: Venn diagram showing that while three proteins overlap between LA myopathy and atrial fibrillation, several others appear to be specific for the disproportionate LA myopathy phenotype (proteins identified using a proteomic analysis in the PROMIS-HFpEF study with validation in the Northwestern University HFpEF cohort). Reproduced with permission from Patel RB et al. [44]

Shah et al. have shown that left atrial (LA) myopathy in HFpEF, defined by reduced LA reservoir strain, is associated with increased pulmonary vascular resistance, decreased peak VO_2_, and poor outcomes [43]. Recently, they defined a unique LA myopathy phenotype in HFpEF in the PROMIS-HFpEF observational study by taking the residuals of linear regression of LA reservoir strain and LV longitudinal strain to define a continuous phenotype that ranges from LA-preserved, to LA/LV-balanced, to LA-predominant (LA myopathy) phenotypes (Fig. 1a; [44]). The LA myopathy phenotype is associated with better LV systolic and diastolic function but worse hemodynamics (lower stroke volume, increased pulmonary artery pressure). In a proteomic analysis in the PROMIS study (with external validation), LA myopathy was associated with increased natriuretic peptides and several novel circulating proteins, which were independent of AF (Fig. 1b, c). Interatrial shunt devices and procedures [45–55] offer a therapeutic option for HFpEF-LA myopathy and therefore could be targeted to these patients if better identified. In addition, automated deep learning algorithms of electrocardiograms and echocardiograms could be trained to identify the LA myopathy HFpEF subtype for further evaluation in clinical trials.

### Genetic subtype

Phase 3 HFpEF clinical trials such as TOPCAT and PARAGON have shown that patients with LVEF > 65%, especially men, do not respond favorably [39, 56] and are particularly resistant to conventional HF therapeutics. Thus, there may be a unique hypercontractile HFpEF phenotype that may or may not be associated with unrecognized cardiomyopathy variants. A recent CHARM trial analysis found that on whole-exome sequencing, 20 of 767 (2.6%) HFpEF patients had pathogenic or likely pathogenic cardiomyopathy rare variants, which was only slightly lower than the rate found in HFrEF (3.5%), and both were much higher than in the general population [57]. While 2.6% may seem trivial, it translates into nearly 80,000 patients in the United States alone given the estimated HFpEF prevalence (over 3 million) in the United States, and is similar to the frequency of transthyretin amyloid cardiomyopathy (~3–4% of HFpEF [58]), for which there is an approved treatment that lowers mortality [59]. In HFpEF, both hypercontractile and hypocontractile (despite preserved global LVEF) subtypes exist, and these HFpEF phenotypes could be treated with myosin inhibitors or activators, respectively, both of which are currently in development [60, 61]. Further investigation of the frequency of rare cardiomyopathy variants in HFpEF and use of deep learning algorithms to train on the electrocardiograms and echocardiograms of HFpEF associated with these variants will be essential to further our understanding of the genetic subtypes of HFpEF.

### Molecular subtype: plasminogen activator inhibitor-1

**Fig. 2:**
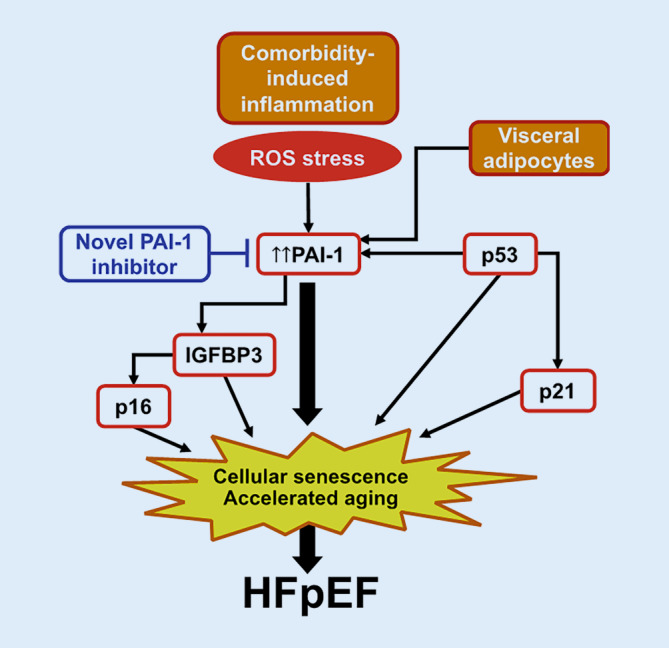
Potential role of plasminogen activator inhibitor‑1 (*PAI‑1*) as a molecular mechanism underlying heart failure with preserved ejection fraction (*HFpEF*). Both comorbidity-induced inflammation (via reactive oxygen species [*ROS*]-induced cellular stress) and visceral adipocytes (common in HFpEF) induce upregulation of PAI‑1 in the circulation. PAI‑1 and related proteins (e.g., insulin-like growth factor binding protein‑3 [*IGFBP3*]) result in cellular senescence and accelerated aging, which may be important risk factors for the development of HFpEF

Plasminogen activator inhibitor (PAI)-1 is one of the only biomarkers found in cross-cohort collaboration studies to be uniquely associated with incident HFpEF but not HFrEF [62]. It has been implicated in aging, senescence, visceral adiposity, and impaired metabolism [63–65]. Activation of PAI‑1 may represent a unique HFpEF subtype characterized by accelerated aging, inflammation, and presence of metabolic comorbidities. Indeed, PAI‑1 is a key protein secreted by metabolically unhealthy visceral adipose tissue, which is found in the vast majority of HFpEF patients. Preclinical studies have identified PAI‑1 as a promoter of senescence (Fig. 2). Clinical deep phenotyping studies in an Old Order Amish kindred found that a rare variant in the gene that encodes for PAI‑1, *SERPINE1*, is associated with PAI‑1 deficiency, increased insulin sensitivity, and longevity [65]. Taken together, these data suggest a central role for PAI‑1 in a potential “accelerated aging” HFpEF phenotype. Studies on the associations between PAI‑1 levels and multi-omics were conducted with HFpEF patients, and these associations could be compared with those in HFrEF and controls. Identification of a unique HFpEF phenotype with elevated PAI‑1 levels is relevant because of the availability of a novel PAI‑1 inhibitor, which is currently being tested in a Phase 2 trial in COVID-19 (clinicaltrials.gov NCT04634799) and could be repurposed for HFpEF. Determining molecular pathways associated with increased PAI‑1 in HFpEF could also identify novel targets, and pathophysiologic abnormalities associated with PAI‑1 in HFpEF could inform endpoints for Phase 2 trials.

## Targeting primary comorbidities in HFpEF

The different clinical manifestations of HFpEF in clinical trials and registries, as well as the failure of generalized medical approaches such as renin–angiotensin–receptor signaling cascade blockers to improve the prognosis in HFpEF cohorts, have emphasized the need for a more differentiated therapeutic strategy. A proposed treatment strategy suggests the identification of leading comorbidities based on the clinical phenotype, which would then guide therapeutic interventions [66]. This approach is in line with current European Society of Cardiology (ESC) guideline recommendations to treat etiologies and cardiovascular and non-cardiovascular comorbidities in HFpEF [67]. However, whether specific treatment of such selected subgroups improves the prognosis in HFpEF remains to be determined. For instance, treating arterial hypertension can reduce the risk for HF, including HFpEF [68]. However, while lowering elevated systolic blood pressure in patients with manifest HFpEF was associated with reduced hospitalization rates it did not lower all-cause or cardiovascular mortality [69].

The majority of HFpEF patients have increased pulmonary artery pressure (pulmonary hypertension [PH]), although in most this is typically pulmonary venous hypertension; however, there are some HFpEF patients who have combined post- and pre-capillary PH. Treatment using specific drugs approved for pulmonary artery hypertension in unselected patients with HFpEF has yielded mixed results and is currently not recommended [70]. Stimulators of the soluble guanyl cyclase (enhancing cGMP-signaling) have been effective in patients with primary pulmonary artery hypertension (riociguat [71]) and also in HFrEF (vericiguat [72]). Yet, vericiguat demonstrated neutral results in HFpEF patients in the VITALITY-HFpEF [73] and SOCRATES-Preserved trials [74]; however, both trials did not differentiate patients without versus with PH.

Dietary restriction in obese patients improves diastolic function [75], and a reduction in calorie intake by ~400 kcal/day increases exercise capacity and quality of life in elderly obese HFpEF patients [76]. Again, the impact on prognosis has yet to be established. Ongoing and future trials of drugs that result in significant weight loss (e.g., GLP‑1 receptor agonists) will hopefully answer the question of whether weight loss improves outcomes in HFpEF. In summary, in patients with HFpEF, treatment of contributing risk factors may or may not be sufficient to treat the cardiac and systemic alterations that they have accumulated from these risk factors.

## Machine learning approaches to the classification of HFpEF

**Fig. 3:**
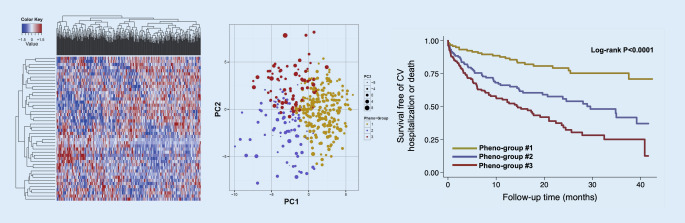
Phenomapping of heart failure with preserved ejection fraction (HFpEF). *Left panel*: hierarchical clustering heatmap of 397 patients with HFpEF (*columns*) and 67 continuous variables (features, *rows*) demonstrating the heterogeneity of HFpEF. *Red* indicates increased and *blue* indicates decreased values. *Middle panel*: principal components analysis showing the clear differentiation of the three identified pheno-groups. *Right panel:* Kaplan–Meier curves for survival free of cardiovascular hospitalization or death among the three pheno-groups. *PC* principal component, *CV* cardiovascular. Reproduced with permission from Shah SJ et al. [7]

**Fig. 4:**
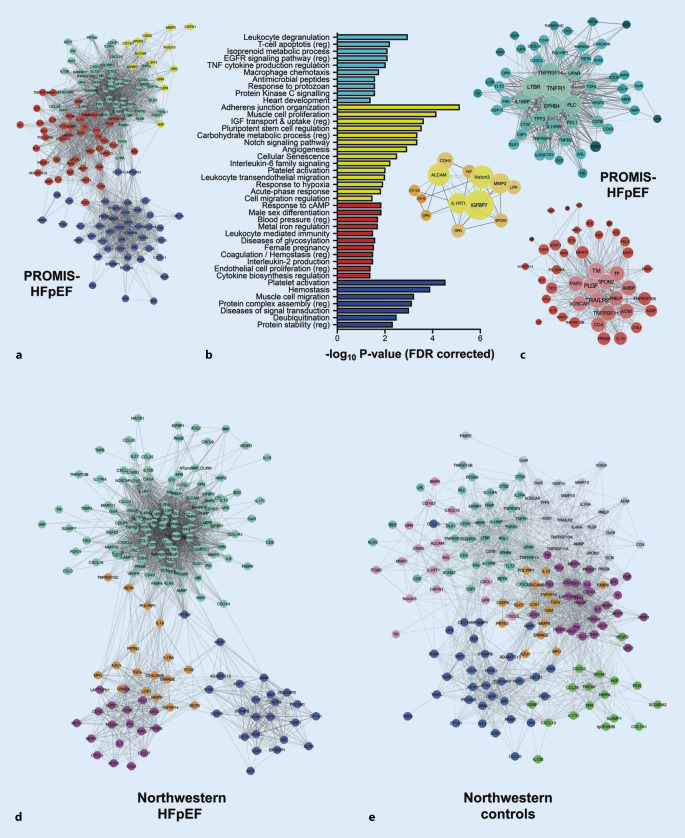
Protein clusters identified by weighted coexpression network analyses in the PROMIS-HFpEF derivation cohort and the Northwestern University validation cohort. The inflammation cluster (*turquoise*) mediated the association between comorbidity burden and markers of elevated left atrial pressure in heart failure with preserved ejection fraction (*HFpEF*) and differentiated HFpEF from controls in an external cohort. **a** Adjacency network map of circulating proteins color-coded by cluster assignment by hierarchical clustering-based nearness or coexpression of proteins. For clarity of presentation, only nodes (proteins) that were assigned to a cluster are shown (*n* = 159/248); the remaining proteins lie on the outer edges of the network map. **b** Overrepresented, nonredundant pathways in each cluster with false discovery rate corrected *p* values. **c** Detailed network maps of proteins in the three clusters that were representative of inflammation (i.e., overrepresentation of ≥2 inflammatory pathways). Node size reflects intracluster connectivity (i.e., the sum of weighted edges [correlations] with all other proteins in the cluster). Node color density reflects the strength of cluster membership. Edge thickness and transparency reflect the adjacency of proteins according to weighted coexpressions. **d** Adjacency network map of circulating proteins in the Northwestern patients with HFpEF in the validation cohort. Clusters with most significant overlap were assigned the same color as the corresponding cluster in the PROMIS-HFpEF cohort. **e** Adjacency network map of circulating proteins in the Northwestern control patients in the validation cohort. Cluster preservation was tested against the Northwestern patients with HFpEF; clusters with significant overlap were assigned the same color as the corresponding cluster in the Northwestern patients with HFpEF. *FDR* false discovery rate, *PROMIS-HFpEF* Prevalence of Microvascular Dysfunction in Heart Failure With Preserved Ejection Fraction, *WCNA* weight coexpression network analysis. Reproduced with permission from Sanders-van Wijk S et al. [9]

As mentioned earlier, multiple studies have now applied data-driven approaches (“phenomapping”) to HFpEF classification. These studies have used machine learning analytic techniques similar to our original phenomapping study and have shown that the HFpEF subtypes identified have different clinical features and outcomes (Fig. 3; [7]). Nonetheless, the study by Shah and colleagues was only the initial “proof of concept” that HFpEF is a truly heterogeneous syndrome (as shown in the heatmap in Fig. 3, left panel), warranting larger-scale, multicenter investigations to advance the science of identifying novel HFpEF subtypes and treatment targets using next-generation phenomics. Importantly, machine learning analysis of deeply phenotyped HFpEF cohorts is only the starting point for further investigation into HFpEF subtypes. While several HFpEF phenomapping studies have been published and have agreed on up to five common HFpEF sub-phenotypes (Fig. 4; [77]), additional research is required to determine the pathophysiological and molecular mechanisms underlying each HFpEF subtype.

As an example, since the initial HFpEF phenomapping study [7], Shah et al. have probed further into the three identified HFpEF subtypes by investigating underlying disease mechanisms and designing and conducting targeted clinical trials in these subtypes, as shown in Table 2. In addition, once HFpEF subtypes have been identified, analytical approaches such as natural language processing and supervised machine learning can be used to assist with automated identification of patients within specific HFpEF subtypes, which will assist with enrollment in future targeted HFpEF clinical trials and targeting of specific therapies to HFpEF subtypes. For example, natural language processing of unstructured electronic health record data has been used to automate the identification of eligible patients for an HFpEF clinical trial (PARAGON; [78]). In addition, large-scale machine learning analyses of national electronic health record claims data have been used to develop a prediction model for amyloidogenic transthyretin cardiomyopathy to improve/automate clinical recognition of this treatable HFpEF subtype [79]. Deep learning machine learning models have also been used for the automated identification of amyloidogenic transthyretin cardiomyopathy using electrocardiography and echocardiography [80].

**Table 2:** Examples of going beyond initial HFpEF phenomapping studies: ongoing follow-up studies of the original three HFpEF pheno-groups identified by Shah et al. [7]

Pheno-group	Follow-up studies
1: Morbid obesity with the BNP deficiency syndrome	STEP-HFpEF: RCT of a GLP1 receptor agonist in obese HFpEF patients
2: Extreme cardiometabolic syndrome	PROMIS-HFpEF: evaluation of impaired coronary flow reserved in HFpEF, and proteomic analysis of comorbidity-inflammation paradigm in HFpEF
3: RV cardio-abdomino-renal syndrome	VICTORY: observational study of exfoliated colonocytes (extracted from stool samples) in hospitalized HFpEF vs. non-HF hospitalized controls to determine whether colonocyte NHE3 (by flow cytometry) is upregulated in HFpEF and whether magnitude of NHE3 upregulation correlates with RV enlargement/dysfunction

*BNP* B-type natriuretic peptide, *RCT* randomized controlled trial, *GLP1* glucagon-like peptide 1, *HFpEF* heart failure with preserved ejection fraction, *NHE3* sodium-hydrogen exchanger‑3, *RV* right ventricular

**Fig. 5:**
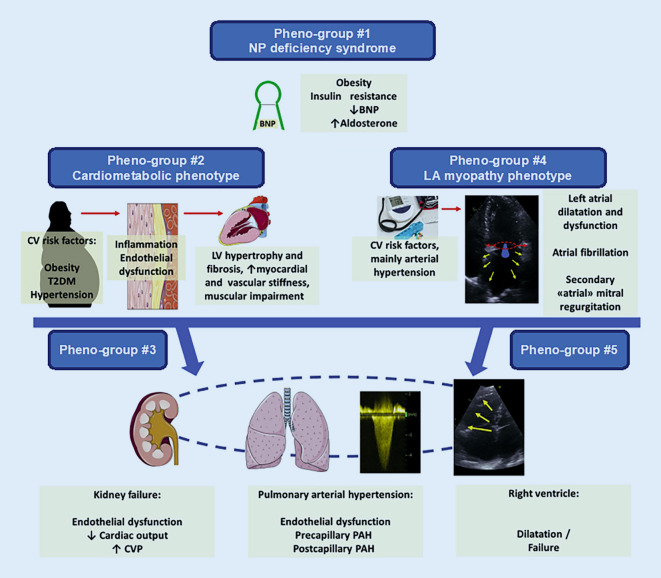
Main heart failure with preserved ejection fraction sub-phenotypes identified across phenomapping studies. *NP* natriuretic peptide, *CV* cardiovascular, *T2DM* type 2 diabetes mellitus, *LV* left ventricular, *CVP* central venous pressure, *PAH* pulmonary arterial hypertension. Reproduced with permission from Galli E et al. [77]

Various types of -omics data can be analyzed with data reduction techniques with subsequent identification of overrepresented biological pathways, which can provide insight into underlying biological mechanisms of HFpEF and its subtypes. This has been done using principal components analysis and weighted coexpression network analyses ([9]; Fig. 5). These techniques reduce high-dimensional data into a low-dimensional data space, summarized by eigenvalues. Eigenvalues can then be used in conventional multivariable regression and mediation analyses to determine associations and perform causal inference with particular HFpEF subtypes.

Despite the rapid rise of machine learning and -omics studies in HF, including HFpEF, there are several challenges to these types of studies that must be considered when evaluating their clinical utility in the classification (sub-phenotyping) of HFpEF and in understanding underlying molecular mechanisms. Table 3 lists key steps in performing machine learning analyses relevant to classification of HFpEF. Table 4 lists various potential problems with these types of studies (e.g., lack of external validation, single timepoint assessments, disease progression bias, cohort bias, feature bias, and publication bias) and offers potential solutions for overcoming these limitations.

**Table 3:** Key steps in the derivation and validation of HFpEF subtypes using unsupervised machine learning (phenomapping) analyses

Step	Details
1	Identify a rich dataset for training (derivation) and a separate, similar dataset for external validation
2	Determine which variables (features) to include in the ML models, evaluate for missingness, perform imputation
3	Data reduction techniques (e.g., PCA) for high-dimensionality data and to account for redundancy
4	Split training dataset into training and test subsets
5	Evaluate a variety of unsupervised ML techniques (or ensemble methods) on the training dataset
6	Cross-validate in the internal test dataset, perform regularization to prevent overfitting
7	Determine optimal (most parsimonious) number of clusters (subtypes) using multiple methods
8	Deploy the model to assign the most probable clusters in the external validation dataset
9	Compare clinical characteristics of pheno-groups (clusters): derivation vs. validation datasets
10	Create a simplified regression model to assign pheno-groups in further validation datasets
11	Follow-up studies to probe disease mechanisms in identified pheno-groups (disease subtypes)

*HFpEF* heart failure with preserved ejection fraction,* ML* machine learning,* PCA* principal components analysis

**Table 4:** HFpEF phenomapping analyses: possible problems and potential solutions

Problem	Explanation	Solution
Lack of external validation	ML models often perform well in the derivation dataset because they are designed to perform well when given a lot of data (features) that are not correlated. However, they may not perform well in an external validation dataset	Always include a validation dataset (preferably external to the derivation dataset) to validate ML models, and plan for this from the design phase of the study
Publication bias	Many ML and -omics studies cannot be explained biologically, or may not validate, both of which create publication biases (results that do not fit known paradigms tend not to be published), limiting clinical applications and future studies	Cloud platforms for data sharing should be implemented which will reduce publication bias and allow future studies to validate or refute our analyses and find meaning in unexplained results. In addition, independent investigators would be able to reanalyze data as new statistical and bioinformatics approaches are developed
Single timepoint measurements	The development of HFpEF likely requires multiple consecutive triggers, creating a dynamically evolving phenotype. Even after clinically overt HFpEF emerges, the underlying molecular phenotype(s) are further evolving with time with disease progression, which will change the circulating proteome/metabolome. Even in healthy individuals, changes in multi-omics over a relatively short time can be strikingly variable. Single timepoint omics data and ML analyses alone will not be able to determine which signals are reactive or causal	When possible, investigators should leverage -omics and other high-dimensional (e.g., imaging) data from serial timepoints, and they should validate any identified signals in multiple cohorts and in orthogonal study types (e.g., transcriptomic analyses, animal studies). Mechanistic experiments on tissues or patient-derived cell lines may also address these challenges
Cohort-driven and feature-driven biases of ML analyses	It is well known that ML studies often suffer from lack of external validation. However, less well known is that the features included in the ML model often drive the identified clusters (subtypes)	Compare unbiased vs. biased selection of features for incorporation in ML models
True pathobiological HFpEF subtypes vs. disease progression HFpEF subtypes	In previous unsupervised ML analyses of HFpEF that sought to identify different HFpEF pathobiological subtypes, often subtypes that represent different stages of HFpEF progression (disease severity) are instead identified	Identification of HFpEF subtypes that reflect disease severity/progression, can still be used to identify and stratify treatment targets, which would be clinically relevant. Upon identification of HFpEF subtypes, investigators should use multivariable analyses to determine whether subtypes are independent of markers of disease severity. Investigators can also use input features that are markers of disease severity prior to inclusion in ML models

*HFpEF* heart failure with preserved ejection fraction, *ML* machine learning

## Examples from HFpEF clinical trials that have informed future phenotype-based treatment

Several ongoing and recently completed HFpEF clinical trials have taken advantage of sub-phenotyping of HFpEF in order to target therapeutics to particular HFpEF subtypes in an attempt to improve chances for HFpEF clinical trial success. For example, in the EMBARK-HFpEF trial (open-label proof-of-concept study of mavacamten [myosin inhibitor] in HFpEF [NCT04766892]), only patients with LVEF > 60%, LV hypertrophy, and elevated biomarkers (elevated natriuretic peptides or troponin) are allowed entry into the trial. These HFpEF patients are most similar to patients with genetic/familiar forms of HCM and therefore are hypothesized to be the patients most likely to benefit (and least likely to have adverse effects) from mavacamten therapy.

In the SERENADE trial (NCT03153111), which is a randomized controlled trial of macitentan (a dual endothelin receptor A and B antagonist) in patients with HFpEF, elevated natriuretic peptides, cardiac remodeling (LVH or LA enlargement), and evidence of pulmonary vascular disease (invasive defined elevation in pulmonary vascular resistance or diastolic pulmonary gradient; or elevated pulmonary artery pressure with evidence of right ventricular dysfunction) are allowed entry into the trial. These patients are hypothesized to be those who will benefit most from macitentan. To further select patients who may benefit, SERENADE included a 4-week placebo run-in phase (to ensure clinical stability) and a 5-week macitentan run-in phase (to exclude patients who develop early fluid retention in response to macitentan). Only patients who qualify for the trial and make it through these two run-in phases were randomized in the trial.

**Fig. 6:**
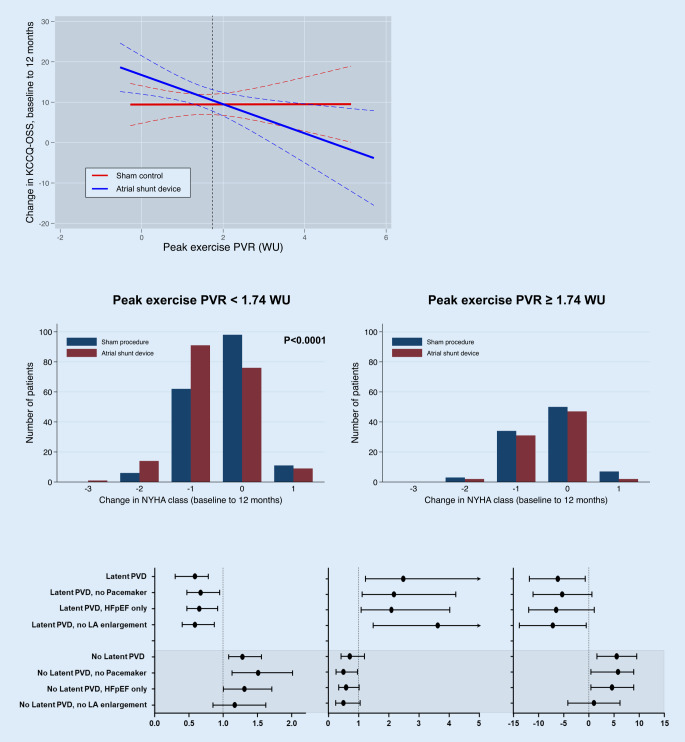
Beneficial effects of interatrial shunt device therapy in patients with heart failure with preserved ejection fraction (*HFpEF*) with peak exercise pulmonary vascular resistance (*PVR*) < 1.74 WU enrolled in the REDUCE LAP-HF II trial. *Top panel*: In the sham control group, there was no association between peak exercise PVR and change in health status (Kansas City Cardiomyopathy Questionnaire overall summary score [KCCQ-OSS]) from baseline to 12 months (all patients improved approximately 10 points). However, in the atrial shunt device-treated patients, those with peak exercise PVR < 1.74 WU improved to a greater extent with the device compared with sham, whereas those with peak exercise PVR ≥ 1.74 WU did worse with the device compared with sham. *Middle panel*: Only HFpEF patients with peak PVR < 1.74 WU experienced a significant improvement in NYHA functional class in the trial.* Bottom panel*: Patients without latent pulmonary vascular disease (*PVD*; i.e., patients with peak exercise PVR < 1.74 WU) and no pacemaker at baseline had the highest win ratio, lowest HF event rate, and greatest improvement in health status in response to the atrial shunt device (compared with sham control). *KCCQ-OSS* Kansas City Cardiomyopathy Questionnaire, *WU* Wood units, *NYHA* New York Heart Association, *LA* left atrial. Reproduced with permission from Borlaug BA et al. [83]

In the recently completed REDUCE LAP-HF II trial ([81]; a phase 3, pivotal, multicenter trial of 626 HFpEF patients randomized 1:1 to an atrial shunt device vs. sham control), all patients were required to have documented evidence of elevated pulmonary capillary wedge pressure (> 25 mm Hg during exercise) on their screening right heart catheterization study. Furthermore, patients with evidence of greater than mild right ventricular dysfunction, greater than mild tricuspid regurgitation, elevated pulmonary vascular resistance (PVR > 3.5 WU), or other reasons to suspect poor outcomes with the device were excluded. Thus, REDUCE LAP-HF II was an enrichment trial (a type of precision medicine trial; [82]). Nevertheless, despite the careful patient selection process, the overall trial results were neutral. However, further post hoc analyses demonstrated that patients with peak exercise pulmonary vascular resistance < 1.74 WU appeared to benefit from the device (Fig. 6; [83]). Thus, future precision medicine trials of atrial shunt devices and procedures in HFpEF may benefit from only including those patients with the ability to vasodilate the pulmonary vasculature during exertion.

## Phenomapping using advanced modalities

Current phenomapping approaches for HFpEF are largely based on clinical data available from demographics and routine clinical parameters such as medical history, medication history, routine blood tests, as well as electrocardiographic and echocardiographic read-outs [7, 15]. Future HFpEF phenomapping studies ideally will be planned prospectively and have already started at various centers across the world (e.g., the US National Institutes of Health HeartShare Study [www.HeartShareStudy.org]). In addition, advanced imaging, e.g., machine learning-based (magnetic resonance) image analysis and deep-learning algorithms for echocardiographic data may provide incremental prognostic value in HFpEF [84, 85]. Metabolic profiling quantifying blood serum metabolites by liquid chromatography–tandem mass spectrometry and proton-nuclear magnetic resonance spectroscopy and evaluation of their response to therapy (e.g., exercise training) has also been used to characterize HFpEF patients [86, 87]. An interesting approach is also to combine medical history and multi-omics analyses (e.g., serum metabolome and gut microbiome in cardiometabolic disease patients) to compute the response to drugs in patient subgroups [88].

## Conclusion

Heart failure with preserved ejection fraction (HFpEF) is a heterogeneous, systemic, multi-organ syndrome that is rising in prevalence and is associated with high morbidity and mortality. While certain therapeutics (e.g., sodium-dependent glucose contransporter‑2 inhibitors) may be beneficial in the large majority of HFpEF patients, most treatments are not well suited for a one-size-fits-all approach. Unsupervised machine learning (phenomapping) combined with -omics analyses is a growing approach to the classification of HFpEF and likely represents the future of HFpEF precision medicine. Nevertheless, investigators and clinicians should understand the potential limitations of such approaches and should augment their initial phenomapping studies of HFpEF with follow-up studies to identify underlying molecular mechanisms with the hope of conducting successful precision medicine trials in the future.

## Declarations

For this article no studies with human participants or animals were performed by any of the authors. All studies mentioned were in accordance with the ethical standards indicated in each case.

## References

[1] Borlaug BA (2020). Evaluation and management of heart failure with preserved ejection fraction. Nat Rev Cardiol.

[2] Redfield MM (2016). Heart failure with preserved ejection fraction. N Engl J Med.

[3] Shah SJ (2016). Phenotype-specific treatment of heart failure with preserved ejection fraction: a multiorgan roadmap. Circulation.

[4] Shah SJ (2020). Research priorities for heart failure with preserved ejection fraction: national heart, lung, and blood institute working group summary. Circulation.

[5] Murphy SP, Ibrahim NE, Januzzi JL (2020). Heart failure with reduced ejection fraction: a review. JAMA.

[6] Katz DH (2017). Phenomapping for the identification of hypertensive patients with the myocardial substrate for heart failure with preserved ejection fraction. J Cardiovasc Transl Res.

[7] Shah SJ (2015). Phenomapping for novel classification of heart failure with preserved ejection fraction. Circulation.

[8] Shah SJ (2019). 20th annual Feigenbaum lecture: Echocardiography for precision medicine-digital biopsy to deconstruct biology. J Am Soc Echocardiogr.

[9] Sanders-van Wijk S (2020). Proteomic evaluation of the comorbidity-inflammation paradigm in heart failure with preserved ejection fraction: results from the PROMIS-HFpEF study. Circulation.

[10] Shah SJ (2018). Prevalence and correlates of coronary microvascular dysfunction in heart failure with preserved ejection fraction: PROMIS-HFpEF. Eur Heart J.

[11] Burke MA (2014). Prognostic importance of pathophysiologic markers in patients with heart failure and preserved ejection fraction. Circ Heart Fail.

[12] Harada D (2020). Different pathophysiology and outcomes of heart failure with preserved ejection fraction stratified by K-means clustering. Front Cardiovasc Med.

[13] Casebeer A (2021). Phenotypic clustering of heart failure with preserved ejection fraction reveals different rates of hospitalization. J Cardiovasc Med.

[14] Schrub F (2020). Heart failure with preserved ejection fraction: A clustering approach to a heterogenous syndrome. Arch Cardiovasc Dis.

[15] Segar MW (2020). Phenomapping of patients with heart failure with preserved ejection fraction using machine learning-based unsupervised cluster analysis. Eur J Heart Fail.

[16] Kobayashi Y (2019). Approaching higher dimension imaging data using cluster-based hierarchical modeling in patients with heart failure preserved ejection fraction. Sci Rep.

[17] Hedman AK (2020). Identification of novel pheno-groups in heart failure with preserved ejection fraction using machine learning. Heart.

[18] Przewlocka-Kosmala M (2019). Contribution of cardiovascular reserve to prognostic categories of heart failure with preserved ejection fraction: a classification based on machine learning. J Am Soc Echocardiogr.

[19] Sanchez-Martinez S (2018). Machine learning analysis of left ventricular function to characterize heart failure with preserved ejection fraction. Circ Cardiovasc Imaging.

[20] Rajkomar A, Dean J, Kohane I (2019). Machine learning in medicine. N Engl J Med.

[21] Deo RC (2015). Machine learning in medicine. Circulation.

[22] Thrasher C, Patterson JH, Fiuzat M (2021). Universal definition and classification of heart failure: pharmacists’ perspective: optimizing guideline-directed medical therapy and educating stakeholders. J Card Fail.

[23] Paulus WJ (2019). Phenotypic persistence in heart failure with preserved ejection fraction. Circ Heart Fail.

[24] Lupon J (2019). Heart failure with preserved ejection fraction infrequently evolves toward a reduced phenotype in long-term survivors. Circ Heart Fail.

[25] Dougherty AH (1984). Congestive heart failure with normal systolic function. Am J Cardiol.

[26] Shah SJ (2016). How to develop and implement a specialized heart failure with preserved ejection fraction clinical program. Curr Cardiol Rep.

[27] Sharma K, Kass DA (2014). Heart failure with preserved ejection fraction: mechanisms, clinical features, and therapies. Circ Res.

[28] Heinzel FR (2020). Left ventricular dysfunction in heart failure with preserved ejection fraction-molecular mechanisms and impact on right ventricular function. Cardiovasc Diagn Ther.

[29] Mishra S, Kass DA (2021). Cellular and molecular pathobiology of heart failure with preserved ejection fraction. Nat Rev Cardiol.

[30] Pieske B (2020). How to diagnose heart failure with preserved ejection fraction: the HFA-PEFF diagnostic algorithm: a consensus recommendation from the Heart Failure Association (HFA) of the European Society of Cardiology (ESC). Eur J Heart Fail.

[31] Bisping E (2014). Targeting cardiac hypertrophy: toward a causal heart failure therapy. J Cardiovasc Pharmacol.

[32] AbouEzzeddine OF (2021). Prevalence of transthyretin amyloid cardiomyopathy in heart failure with preserved ejection fraction. JAMA Cardiol.

[33] McHugh K (2019). Heart failure with preserved ejection fraction and diabetes: JACC state-of-the-art review. J Am Coll Cardiol.

[34] van Heerebeek L (2008). Diastolic stiffness of the failing diabetic heart: importance of fibrosis, advanced glycation end products, and myocyte resting tension. Circulation.

[35] Lejeune S (2021). Diabetic phenotype and prognosis of patients with heart failure and preserved ejection fraction in a real life cohort. Cardiovasc Diabetol.

[36] Maack C (2018). Heart failure and diabetes: metabolic alterations and therapeutic interventions: a state-of-the-art review from the Translational Research Committee of the Heart Failure Association-European Society of Cardiology. Eur Heart J.

[37] Unger ED (2016). Association of chronic kidney disease with abnormal cardiac mechanics and adverse outcomes in patients with heart failure and preserved ejection fraction. Eur J Heart Fail.

[38] Odajima S (2022). Efficacy of Renin-angiotensin-aldosterone-system inhibitors for heart failure with preserved ejection fraction and left ventricular hypertrophy -from the KUNIUMI Registry Acute Cohort. J Cardiol.

[39] Solomon SD (2019). Angiotensin-neprilysin inhibition in heart failure with preserved ejection fraction. N Engl J Med.

[40] Solomon SD (2012). The angiotensin receptor neprilysin inhibitor LCZ696 in heart failure with preserved ejection fraction: a phase 2 double-blind randomised controlled trial. Lancet.

[41] Anker SD (2021). Empagliflozin in heart failure with a preserved ejection fraction. N Engl J Med.

[42] Yusuf S (2003). Effects of candesartan in patients with chronic heart failure and preserved left-ventricular ejection fraction: the CHARM-preserved trial. Lancet.

[43] Freed BH (2016). Prognostic utility and clinical significance of cardiac mechanics in heart failure with preserved ejection fraction: importance of left atrial strain. Circ Cardiovasc Imaging.

[44] Patel RB (2021). Disproportionate left atrial myopathy in heart failure with preserved ejection fraction among participants of the PROMIS-HFpEF study. Sci Rep.

[45] Berry N (2020). Transcatheter interatrial shunt device for the treatment of heart failure: rationale and design of the pivotal randomized trial to REDUCE elevated left atrial pressure in patients with heart failure II (REDUCE LAP-HF II). Am Heart J.

[46] Hanff TC (2019). Assessment of predictors of left atrial volume response to a transcatheter interatrial shunt device (from the REDUCE LAP-HF trial). Am J Cardiol.

[47] Feldman T (2016). Transcatheter interatrial shunt device for the treatment of heart failure: rationale and design of the randomized trial to REDUCE elevated left atrial pressure in heart failure (REDUCE LAP-HF I). Circ Heart Fail.

[48] Wessler J (2018). Impact of baseline hemodynamics on the effects of a transcatheter Interatrial shunt device in heart failure with preserved ejection fraction. Circ Heart Fail.

[49] Feldman T (2018). Transcatheter Interatrial shunt device for the treatment of heart failure with preserved ejection fraction (REDUCE LAP-HF I [reduce elevated left atrial pressure in patients with heart failure]): a phase 2, randomized, sham-controlled trial. Circulation.

[50] Obokata M (2019). Effects of interatrial shunt on pulmonary vascular function in heart failure with preserved ejection fraction. J Am Coll Cardiol.

[51] Griffin JM (2020). Impact of interatrial shunts on invasive hemodynamics and exercise tolerance in patients with heart failure. J Am Heart Assoc.

[52] Hasenfuss G (2015). Rationale and design of the reduce elevated left atrial pressure in patients with heart failure (reduce LAP-HF) trial. J Card Fail.

[53] Kaye D (2014). Effects of an interatrial shunt on rest and exercise hemodynamics: results of a computer simulation in heart failure. J Card Fail.

[54] Shah S, Feldman JT, Massaro J (2019). Evaluating treatment effect of transcatheter Interatrial shunt device using heart failure event rates-reply. JAMA Cardiol.

[55] Shah SJ (2018). One-year safety and clinical outcomes of a transcatheter Interatrial shunt device for the treatment of heart failure with preserved ejection fraction in the reduce elevated left atrial pressure in patients with heart failure (REDUCE LAP-HF I) trial: a randomized clinical trial. JAMA Cardiol.

[56] Solomon SD (2016). Influence of ejection fraction on outcomes and efficacy of spironolactone in patients with heart failure with preserved ejection fraction. Eur Heart J.

[57] Povysil G (2020). Assessing the role of rare genetic variation in patients with heart failure. JAMA Cardiol.

[58] Kazi DS (2020). Cost-effectiveness of tafamidis therapy for transthyretin amyloid cardiomyopathy. Circulation.

[59] Maurer MS (2018). Tafamidis treatment for patients with transthyretin amyloid cardiomyopathy. N Engl J Med.

[60] Bond LM (2013). Small-molecule inhibitors of myosin proteins. Future Med Chem.

[61] Kaplinsky E, Mallarkey G (2018). Cardiac myosin activators for heart failure therapy: focus on omecamtiv mecarbil. Drugs Context.

[62] de Boer RA (2018). Association of cardiovascular biomarkers with incident heart failure with preserved and reduced ejection fraction. JAMA Cardiol.

[63] Vaughan DE (2017). Plasminogen activator inhibitor-1 is a marker and a mediator of senescence. Arterioscler Thromb Vasc Biol.

[64] Sun T (2019). PAI-1 contributes to homocysteine-induced cellular senescence. Cell Signal.

[65] Khan SS (2017). A null mutation in SERPINE1 protects against biological aging in humans. Sci Adv.

[66] Senni M (2014). New strategies for heart failure with preserved ejection fraction: the importance of targeted therapies for heart failure phenotypes. Eur Heart J.

[67] McDonagh TA (2022). 2021 ESC Guidelines for the diagnosis and treatment of acute and chronic heart failure: developed by the Task Force for the diagnosis and treatment of acute and chronic heart failure of the European Society of Cardiology (ESC). With the special contribution of the Heart Failure Association (HFA) of the ESC. Eur J Heart Fail.

[68] Schocken DD (2008). Prevention of heart failure: a scientific statement from the American Heart Association Councils on Epidemiology and Prevention, Clinical Cardiology, Cardiovascular Nursing, and High Blood Pressure Research; Quality of Care and Outcomes Research Interdisciplinary Working Group; and Functional Genomics and Translational Biology Interdisciplinary Working Group. Circulation.

[69] Kawano H (2019). Effects of blood pressure lowering in patients with heart failure with preserved ejection fraction: a systematic review and meta-analysis. Hypertens Res.

[70] Vachiéry J-L (2019). Pulmonary hypertension due to left heart disease. Eur Respir J.

[71] Ghofrani HA (2013). Riociguat for the treatment of pulmonary arterial hypertension. N Engl J Med.

[72] Gheorghiade M (2015). Effect of vericiguat, a soluble guanylate cyclase stimulator, on Natriuretic peptide levels in patients with worsening chronic heart failure and reduced ejection fraction: the SOCRATES-REDUCED randomized trial. JAMA.

[73] Armstrong PW (2020). Effect of vericiguat vs placebo on quality of life in patients with heart failure and preserved ejection fraction: the VITALITY-HFpEF randomized clinical trial. JAMA.

[74] Pieske B (2017). Vericiguat in patients with worsening chronic heart failure and preserved ejection fraction: results of the SOluble guanylate Cyclase stimulatoR in heArT failurE patientS with PRESERVED EF (SOCRATES-PRESERVED) study. Eur Heart J.

[75] Hammer S (2008). Prolonged caloric restriction in obese patients with type 2 diabetes mellitus decreases myocardial triglyceride content and improves myocardial function. J Am Coll Cardiol.

[76] Kitzman DW (2016). Effect of caloric restriction or aerobic exercise training on peak oxygen consumption and quality of life in obese older patients with heart failure with preserved ejection fraction: a randomized clinical trial. JAMA.

[77] Galli E (2021). Phenomapping heart failure with preserved ejection fraction using machine learning cluster analysis: prognostic and therapeutic implications. Heart Fail Clin.

[78] Jonnalagadda SR (2017). Text mining of the electronic health record: an information extraction approach for automated identification and Subphenotyping of HFpEF patients for clinical trials. J Cardiovasc Transl Res.

[79] Ahsan H (2021). A machine learning model of medical claims data for identifying patients at risk for wild-type transthyretin amyloid cardiomyopathy. Nat Commun.

[80] Goto S (2020). Artificial intelligence-enabled, fully automated detection of cardiac amyloidosis using electrocardiograms and echocardiograms.

[81] Shah SJ (2022). Atrial shunt device for heart failure with preserved and mildly reduced ejection fraction (REDUCE LAP-HF II): a randomised, multicentre, blinded, sham-controlled trial. Lancet.

[82] Shah SJ (2017). Innovative clinical trial designs for precision medicine in heart failure with preserved ejection fraction. J Cardiovasc Transl Res.

[83] Borlaug BA (2022). Latent pulmonary vascular disease may alter the response to therapeutic atrial shunt device in heart failure. Circulation.

[84] Kucukseymen S (2021). Noncontrast cardiac magnetic resonance imaging predictors of heart failure hospitalization in heart failure with preserved ejection fraction. J Magn Reson Imaging.

[85] Pandey A (2021). Deep-learning models for the echocardiographic assessment of diastolic dysfunction. JACC Cardiovasc Imaging.

[86] Zordoky BN (2015). Metabolomic fingerprint of heart failure with preserved ejection fraction. PLoS One.

[87] Bahls M (2019). Heterogeneous metabolic response to exercise training in heart failure with preserved ejection fraction. J Clin Med.

[88] Forslund SK (2021). Combinatorial, additive and dose-dependent drug-microbiome associations. Nature.

